# Chemogenetics Reveal an Anterior Cingulate–Thalamic Pathway for Attending to Task-Relevant Information

**DOI:** 10.1093/cercor/bhaa353

**Published:** 2020-11-30

**Authors:** Emma J Bubb, John P Aggleton, Shane M O’Mara, Andrew J D Nelson

**Affiliations:** 1 School of Psychology, Cardiff University, Wales CF10 3AT, UK; 2 Institute of Neuroscience, Trinity College Dublin D02 PN40, Ireland

**Keywords:** anterior cingulate cortex, anterior thalamic nuclei, attentional-set formation, DREADDs, extradimensional set-shift

## Abstract

In a changing environment, organisms need to decide when to select items that resemble previously rewarded stimuli and when it is best to switch to other stimulus types. Here, we used chemogenetic techniques to provide causal evidence that activity in the rodent anterior cingulate cortex and its efferents to the anterior thalamic nuclei modulate the ability to attend to reliable predictors of important outcomes. Rats completed an attentional set-shifting paradigm that first measures the ability to master serial discriminations involving a constant stimulus dimension that reliably predicts reinforcement (intradimensional-shift), followed by the ability to shift attention to a previously irrelevant class of stimuli when reinforcement contingencies change (extradimensional-shift). Chemogenetic disruption of the anterior cingulate cortex (Experiment 1) as well as selective disruption of anterior cingulate efferents to the anterior thalamic nuclei (Experiment 2) impaired intradimensional learning but facilitated 2 sets of extradimensional-shifts. This pattern of results signals the loss of a corticothalamic system for cognitive control that preferentially processes stimuli resembling those previously associated with reward. Previous studies highlight a separate medial prefrontal system that promotes the converse pattern, that is, switching to hitherto inconsistent predictors of reward when contingencies change. Competition between these 2 systems regulates cognitive flexibility and choice.

## Introduction

In a dynamic world, the ability to engage in adaptive behaviors is critical to an organism’s survival. This includes deciding when to select items that resemble consistently rewarded stimuli and when to switch to previously irrelevant stimulus types. The ability to disengage from previously rewarded response strategies depends on the integrity of prefrontal cortex. Consequently, prefrontal damage in humans, marmosets, and rats causes response perseveration and a failure to switch when contingencies change (Milner 1963; Dias et al. 1996a, 1996b; Birrell and Brown 2000; Ng et al. 2007; Nyhus and Barceló 2009). Further research with rats highlights how interactions between medial prefrontal cortex and subcortical sites support this form of behavioral flexibility (Block et al. 2007; Baker and Ragozzino 2014; Dolleman-Van Der Weel et al. 2019). However, until recently, there has been little progress in identifying the neural circuits in rodents that support the opposing attentional mechanism, that is, the preferential processing of stimuli resembling those previously associated with important outcomes.

The attentional set-shifting paradigm captures both potentially conflicting cognitive processes. In this task, animals show accelerated learning over successive discriminations by attending to a common stimulus dimension (intradimensional-set [ID]) (Mackintosh 1975). This learnt bias to a specific category (attentional-set) is further revealed when the stimulus being rewarded switches to a qualitatively different category. Now, additional trials, the “shift-cost,” are required to solve this extradimensional-shift (ED) (Birrell and Brown 2000; Chase et al. 2012). This “cost” is increased by medial prefrontal lesions (Birrell and Brown 2000). In contrast, lesions in the rodent anterior thalamic nuclei (ATN) disrupt animals’ ability to form an attentional-set, as revealed by impaired ID-set performance, but paradoxically, when required to solve discriminations involving hitherto irrelevant stimulus dimensions, lesion animals not only outperform controls, but display a shift-benefit (Wright et al. 2015).

The implication is that the ATN are vital for attending to reliable predictors of reinforcement, driving attentional-set formation at the expense of ED-shifts. This bias is then lost following ATN lesions, resulting in heightened attention to inconsistent predictors of reward. This interpretation is supported by both functional imaging and clinical data in humans (de Bourbon-Teles et al. 2014; Leszczyński and Staudigl 2016). The resulting dissociation between the effects of prefrontal lesions (impaired ED-shift) and ATN lesions (facilitated ED-shift) points to a distinct role for the ATN within corticothalamic circuits supporting attentional processes. A key question remains, therefore, with which cortical sites might the ATN act to support these processes?

A potential partner is the anterior cingulate cortex (ACC). The ATN are densely interconnected with the ACC (Shibata 1993; Shibata and Naito 2005; Wright et al. 2013), and there is evidence that the rodent ACC contributes to attentional processing (Ng et al. 2007; Koike et al. 2016). For example, rats with ACC lesions oversample never-reinforced stimuli and appear more distracted by irrelevant information (Ragozzino and Rozman 2007; Newman and McGaughy 2011). To test this potential partnership, we first virally expressed the inhibitory hM4Di DREADD receptor in dorsal ACC. Rats then received behavioral assays explicitly designed to contrast attentional-set formation and ED set-shifting (Chase et al. 2012; Lindgren et al. 2013). For these assays, the rats first received a series of 2-choice discriminations based on either odors or digging media and underwent a series of 4, consecutive ID-shifts, during which one dimension (e.g., odor) is consistently rewarded, whereas the other dimension (e.g., media) remains irrelevant. Next, rats experienced an ED-shift in which the previously irrelevant dimension is now rewarded. Subsequently, the rats performed a second ED-shift task in which spatial position became, for the first time, relevant (Wright et al. 2015). Last, we interrogated the effects of DREADD activation by measuring expression of the immediate-early gene, c-*fos*, in the ACC and related cortical and subcortical targets. This gene was selected as it can provide an indirect marker of neuronal activity (Chaudhuri 1997; Tischmeyer and Grimm 1999; Guzowski et al. 2005). Rats were, therefore, placed in a novel environment in order to increase c-*fos* expression in sites including the ACC and ATN (Zhu et al. 1995; Vann et al. 2000; Wirtshafter 2005).

Experiment 2 tested the specific hypothesis that interactions between the ACC and ATN support these attentional processes. Taking advantage of the anterograde transport of an adeno-associated virus expressing the inhibitory hM4Di DREADD receptor, coupled with localized infusions of the ligand within the ATN, we examined the effects of chemogentically disrupting ACC terminations within ATN on the same attentional set-shifting tasks.

## Materials and Methods

### Subjects

All experiments involved experimentally naïve, male Lister Hooded rats (Envigo, Bicester). The rats were housed in pairs in a temperature-controlled room. At the time of surgery, the rats in Experiment 1 (*n* = 22) weighed between 290 and 331 g, those in Experiment 2 (*n* = 18) weighed between 296 and 328 g. Lighting was kept on a 12-h light/dark cycle, light from 0800 to 2000. During behavioral testing, all animals were food restricted to maintain at least 85% of their free-feeding body weight, whereas water was available ad libitum. All experiments were carried out in accordance with UK Animals (Scientific Procedures) Act, 1986 and European Union (EU) directive (2010/63/EU) as well as local ethical approval from Cardiff University.

### Surgical Procedures

All rats were anesthetized with isoflurane (4% induction, 2% thereafter). Next, each rat was placed in a stereotaxic frame (David Kopf Instruments), with the skull flat (Experiment 1) or with the incisor bar set at +5.0 to the horizontal plane (Experiment 2). For analgesic purposes, Lidocaine was administered topically to the scalp (0.1 mL of 20 mg/mL solution; B. Braun) and meloxicam was given subcutaneously (0.06 mL of 5 mg/mL solution, Boehringer Ingelheim Ltd). A craniotomy was then made directly above the target region and the dura cut to expose the cortex.

In both experiments, the experimental group received injections of an adeno-associated virus expressing the inhibitory hM4Di DREADD receptor into the ACC, whereas control animals received injections of the same virus not expressing the DREADD receptor. Injections were made via a 10 μL Hamilton syringe (Bonaduz) attached to a moveable arm mounted to the stereotaxic frame. The injections were controlled by a microprocessor (World Precision Instruments) set to a flow rate of 0.1 μL/min, and the needle left in situ for a further 5 min to allow for diffusion of the bolus.

In Experiment 1, the experimental group (*n* = 12) received injections of AAV5-CaMKIIa-hM4Di-mCherry (titer 4.4 × 10^12 GC/mL; Addgene) and the control group (*n* = 10) received injections of AAV5-CaMKIIa-EGFP (titer 4.3 × 10^12 GC/mL; Addgene). The injection coordinates and volumes for the 3 injections made into the ACC were as follows: 1) 0.35 μL at AP +1.9, ML +/−0.8, DV −1.2; 2) 0.7 μL at AP +1.0, ML +/−0.8, DV −1.6; and 3) 0.7 μL at AP +0.1, ML +/−0.8, DV −1.6. AP coordinates were taken from bregma, ML coordinates were taken from the sagittal sinus, and DV coordinates were taken from dura.

In Experiment 2, 10 animals received injections of AAV5-CaMKIIa-hM4Di-mCherry (titer 9.5 × 10^12 GC/mL; Addgene), whereas 8 animals received injections of a non-DREADD expressing viral control AAV5-CaMKIIa-EGFP (titer 4.3 × 10^12 GC/ml; Addgene). All animals received 3 viral injections in the ACC in each hemisphere as follows (skull at +5.0 mm to horizontal plane): 1) 0.35 μL at AP +2.1, ML +/−0.8, DV −1.2; 2) 0.65 μL at AP +1.4, ML +/−0.8, DV −1.6; and 3) 0.65 μL at AP +0.7, ML +/−0.8, DV −1.6. The injection volumes were as Experiment 1.

To target anterior cingulate efferents to the ATN, all animals in Experiment 2 were also implanted with guide cannulae aimed at the ATN. A craniotomy was drilled in each hemisphere and bilateral guide cannulae (Plastics One) were implanted (26 gauge, cut to a length of 5.4 mm, center to center distance of 2 mm) in the ATN at the following coordinates: AP: −0.1, ML: +/−1.0, DV: −4.6 (mm from bregma). Cannulae were held in place by bone cement (Zimmer Biomet) and anchored to the skull with 4 fixing screws (Precision Technology Supplies). Removable obturators (Plastic One) were inserted into the guide cannulae to prevent the cannulae from blocking.

For all animals (Experiments 1 and 2), the surgical site was closed using sutures and the analgesic bupivacaine (Pfizer) was injected between the suture sites. A topical antibiotic powder Clindamycin (Pfizer) was then applied to the site. Animals were administered a subcutaneous injection of glucose–saline (5 mL) for fluid replacement before being placed in a recovery chamber until they regained consciousness. Animals were monitored carefully postoperatively with food available ad libitum until they had fully recovered, with behavioral testing commencing 14 days after surgery.

### Behavior

#### Apparatus

All pretraining and testing took place in a black Perspex arena that measured 69.5 cm long, 40.5 cm wide and 18.6 cm tall (Wright et al. 2015). One end of the testing arena comprised 2 individual chambers that were separated from the remaining open space by black Perspex panels that could be removed by the experimenter to allow access. Each of the 3 compartments had a hinged, transparent Perspex lid. In each of the 2 smaller compartments was a circular glass pot (75 mm diameter, 45 mm height) that contained the digging media. Against the opposite wall, in the larger compartment, there was an identical glass pot containing water.

#### Pretraining

Two weeks after surgery, animals underwent 3 days of pretraining. On the first day of pretraining animals were habituated to the arena for 10 min, with access to all 3 chambers and no glass pots present. On the second day of pretraining, all 3 glass pots were in place, with the 2 glass pots in the smaller chambers filled with bedding sawdust. Panels were removed providing access to alternating chambers across trials to prevent the formation of a side bias. On the first trial, half a Cheerio (Nestle) was placed on top of the sawdust, and it was progressively buried in subsequent trials to teach animals to dig for the food reward. This ability was typically acquired within 10 trials. The day before testing took place, animals were preexposed to the test stimuli (Table 1). Each odor was presented with bedding sawdust and each digging media was presented without odor. Animals retrieved half a buried cheerio from each pot of sawdust laced with odor and each pot of odorless digging media, once in each chamber.

**Table 1:** Depiction of a possible order of stimulus pairings in the attentional set-shifting task

Discrimination	Rewarded dimension	Rewarded stimuli	Unrewarded stimuli
SD	Media	**Coarse tea**	Fine tea
CD	Media	**Coarse tea** + cinnamon	Fine tea + ginger
		**Coarse tea** + ginger	Fine tea + cinnamon
ID1	Media	**Coarse cork** + tarragon	Fine cork + fenugreek
		**Coarse cork** + fenugreek	Fine cork + tarragon
ID2	Media	**Wood shavings** + marjoram	Wood chip + sage
		**Wood shavings** + sage	Wood chip + marjoram
ID3	Media	**Short cigarette filters** + cumin	Long cigarette filters + dill
		**Short cigarette filters** + dill	Long cigarette filters + cumin
ID4	Media	**Beanbag filler** + mint	Polystyrene + turmeric
		**Beanbag filler** + turmeric	Polystyrene + mint
ED	Odor	Confetti + **cloves**	Oregano + shredded paper
		Shredded paper + **cloves**	Oregano + confetti
REV	Odor	Confetti + **oregano**	Cloves + shredded paper
		Shredded paper + **oregano**	Cloves + confetti

Note: Stimuli from the relevant dimension, which consistently signify reward location, are in bold. In this example, digging media is the first dimension relevant to the location of the buried food reward. From the ED stage onward, odor is the relevant dimension. Stimuli are always paired as shown, but the discrimination in which animals encounter them is counterbalanced. The first dimension to be rewarded is also counterbalanced across animals.

#### DREADD Activation

Three weeks after surgery, behavioral testing on the attentional set-shifting task began. Prior to testing, the DREADDS were activated by clozapine (Gomez et al. 2017; Tan et al. 2020) either by systemic injection (Experiment 1) or intracranial infusion (Experiment 2).

In Experiment 1, clozapine dihydrochloride (Hello Bio) was dissolved in sterile saline. Twenty minutes before the test began, all rats received an intraperitoneal (I.P.) injection of clozapine dihydrochloride at a dose of 4 mg/kg (as salt).

In Experiment 2, clozapine dihydrochloride (Hello Bio) was dissolved in sterile saline at a dose of 1 μg/μL (as salt). Fifteen minutes prior to testing, rats were lightly restrained, the obturators removed, and 33-gauge stainless steel infusion cannulae (Plastic One) that projected 2.0 mm beyond the tip of the guide cannulae were inserted. Each pair of infusion cannula was connected to two 5 μL Hamilton syringes (Bonaduz) mounted on 2 infusions pumps (Harvard Apparatus Ltd). A volume of 0.25 μL per hemisphere was infused over 1 min. The infusion cannulae were left in situ for a further 1 min to allow absorption of the bolus. The infusion cannulae were then removed and the obturators replaced.

#### Behavioral Training: Attentional Set-Shifting Task

Following activation of the DREADDS, the rats received a single test session in the arena. The glass pots in the 2 smaller compartments of the arena were filled with different stimuli pairs (Table 1). Only 1 pot contained the buried food reward (half a Cheerio, Nestle). Animals encountered a sequence of discriminations requiring them to learn to select the correct stimulus in order to retrieve the food reward, before beginning the next discrimination.

At the beginning of each trial, the dividing panels were removed allowing the animal access to the 2 smaller compartments. The compartment of the correct pot was pseudorandomly allocated in each trial. If the animal dug in the correct pot, defined as breaking the surface of the digging media with paws or nose, it could retrieve the reward. For the first 4 trials of each discrimination, the animal was allowed access to the correct compartment to retrieve the reward following an initial dig in the incorrect pot. Thereafter, if the animal dug in the incorrect pot, access to the correct compartment was blocked. The intertrial interval lasted approximately 5 s during which time the pots were rebaited. Once the animal had acquired a discrimination, quantified by 6 consecutive correct digs, it moved on to the next discrimination.

There were 8 consecutive discriminations (Table 1). For the initial 6 discriminations, one dimension, for example, type of digging media, consistently predicted reinforcement. The session began with a “simple discrimination” (SD) where 2 distinct digging medias were discriminated. Next, in the “compound discrimination” (CD), the same digging medium continued to be rewarded but stimuli from another dimension (odors) were added to create 2 different discrimination types (Table 1). For the next 4 discriminations (ID1–4) the rewarded dimension remained constant (i.e., digging media), but the particular stimuli changed from discrimination to discrimination. Throughout, the other dimension (odor) was present, but no individual odor consistently predicted reward. For the ED-shift, rats were now required to switch dimensions to solve the discrimination, for example, from a particular digging medium to a particular odor (Table 1). Finally, rats received a “reversal” (REV) in which the particular pair of stimuli in the rewarded dimension had their reward contingencies reversed (Table 1). All testing contingencies were counterbalanced across animals in each group, so that half began with odor as the rewarded dimension.

#### Second Extradimensional-Shift (Spatial)

Approximately 2 weeks after the first behavioral test, rats completed a second series of discriminations. These took place in the same apparatus and followed the same procedure but comprised 4 discriminations (Table 2). Prior to this additional test, DREADDS were activated as described above. First, the animals acquired a CD followed by an ID. The rewarded dimension in the first 2 discriminations (CD, ID) matched that last rewarded in the first behavioral study (i.e., in the ED and REV). Next, there was an extradimensional-shift based on the spatial location of the digging pots (ED_spatial_, Table 2). Here, for the first time, whether the pot was located in the left or the right chamber became the critical rewarded feature. Both odor and type of digging media were now irrelevant. Following acquisition of this spatial discrimination, the left/right contingencies were reversed (REV_spatial_, Table 2).

**Table 2:** Possible order of stimulus pairings in the second attentional set-shifting task

Discrimination	Rewarded dimension	Rewarded stimuli	Unrewarded stimuli
CD	Odor	**Paprika** + short wire	Coriander + long wire
		**Paprika** + long wire	Coriander + short wire
ID	Odor	**Lemongrass** + buttons	Nutmeg + beads
		**Lemongrass** + beads	Nutmeg + buttons
ED_spatial_	Spatial location	Lemongrass + buttons (**left chamber**)	Nutmeg + beads (right chamber)
		Lemongrass + beads (**left chamber**)	Nutmeg + buttons (right chamber)
		Nutmeg + beads (**left chamber**)	Lemongrass + buttons (right chamber)
		Nutmeg + buttons (**left chamber**)	Lemongrass + beads (right chamber)
REV_spatial_	Spatial location	Lemongrass + buttons (**right chamber**)	Nutmeg + beads (left chamber)
		Lemongrass + beads (**right chamber**)	Nutmeg + buttons (left chamber)
		Nutmeg + beads (**right chamber**)	Lemongrass + buttons (right chamber)
		Nutmeg + buttons (**right chamber**)	Lemongrass + beads (right) chamber)

Note: Depiction of one possible order of stimulus pairings in the additional set-shifting task. Here, odor is the first dimension relevant to the location of the buried food reward as it was the most recently rewarded dimension from the prior behavioral test (ED and REV, see Table 1). From the ED_spatial_ stage onward, the location of the pot (right or left) is the relevant dimension. Stimuli were paired as shown, but the discrimination in which animals encounter them is counterbalanced between animals. The first spatial location (right or left) to be rewarded was counterbalanced across animals.

#### c-fos Expression (Experiment 1 Only)

One week after the completion of behavioral testing, all rats received an I.P. injection of clozapine dihydrochloride (4 mg/kg as salt; HelloBio) to activate DREADDS in the experimental group. Each rat was placed individually in a cage in a dark holding room for 20 min, followed by 2 novel environments, each for 15 min. The first was a large square open field arena measuring 100 cm long, 100 cm wide, and 45 cm tall, which was filled with bedding sawdust and 6 novels objects. The second was a bow–tie maze (Albasser et al. 2010), which measured 120 cm long, 50 cm wide, and 50 cm tall, which was filled with bedding sawdust and food rewards (Cheerios, Nestle). Rats were then returned to the dark holding room for 90 min, to allow for neuronal Fos expression (Bisler et al. 2002) before perfusion.

### Histology

Immediately prior to perfusion, animals received an I.P. injection of a lethal dose of sodium pentobarbital (2 mL/kg, Euthatal, Marial Animal Health) and were transcardially perfused with 0.1 M phosphate-buffered saline (PBS), followed by 4% paraformaldehyde in 0.1 M PBS (PFA). Brains were removed, postfixed in PFA for 2 h, and then placed in 25% sucrose solution for 24 h at room temperature on a stirring plate.

Brains were cut into 40-μm coronal sections using a freezing microtome (8000 sledge microtome, Bright Instruments) and a series of 1 in 4 sections was collected in PBS for fluorescence analysis (Experiments 1 and 2) and Fos analysis (Experiment 1). To verify cannulae placements, an additional series was collected for cresyl staining (Experiment 2).

#### DREADDs Expression Immunohistochemistry

Immunohistochemistry was carried out on the tissue to enhance the fluorescence signal of mCherry (DREADD groups). The first series of sections was transferred from PBS into a blocking solution of 5% normal goat serum (NGS) in PBS with Tritonx-1000 (PBST) and incubated for 1 h. The sections were then transferred into the primary antibody solution of rabbit-anti-mCherry (Abcam) at a dilution of 1:1000 in PBST with 1% NGS and incubated for 24 h. Sections were washed 4 times in PBST and transferred to a secondary antibody solution of goat-anti-rabbit (Dylight Alexa flour 594, Vector Laboratories) or at a dilution of 1:200 at PBST. From this point onwards the sections were kept in the dark. Sections were incubated for 1 h before being washed 3 times in PBS. Sections were mounted onto gelatine-subbed glass slides and allowed to dry overnight before being immersed in xylene and coverslipped using DPX (Thermo Fisher Scientific). All incubations were on a stirring plate at room temperature and all washes were for 10 min. Virus expression was analyzed using a fluorescent Leica DM5000B microscope with a Leica DFC310 FX camera. Images were collected from the ACC, selected efferent targets, and a comparison cortical area, secondary somatosensory cortex.

#### Fos Immunohistochemistry

Sections were washed 4 times in PBS, once in a peroxidase block (0.3% hydrogen peroxidase in PBST) and 4 times in PBST. The sections were then transferred to a blocking solution of 3% NGS in PBST and incubated for 1 h. The sections were then transferred to a primary antibody solution of rabbit-anti-c-*fos* (Millipore) at a dilution of 1:5000 in PBST and incubated for 10 min, followed by 48 h at 4°C in a refrigerator. Sections were washed 4 times in PBST and transferred to a secondary antibody solution of goat-anti-rabbit (Vector Laboratories) at a dilution of 1:200 in 1.5% NGS in PBST. Sections were incubated for 2 h before being washed 4 times in PBST and transferred to an avidin/biotinylated enzyme complex (Vectastain ABC HRP kit, Vector Laboratories) in PBST for 1 h. Sections were washed 4 times in PBST and twice in a Tris buffer (0.6% trisma base in distilled water). Sections were then immersed in a DAB solution (DAB peroxidase HRP substrate kit, Vector Laboratories) for 1–2 min before the reaction was stopped with cold PBS. The sections were mounted onto gelatin-subbed glass slides and allowed to dry overnight before being immersed in xylene and coverslipped using DPX. All incubations were on a stirring plate at room temperature and all washes were for 10 min.

#### Fos: Cell Counting, Regions of Interest, and Analyses

Fos-positive cells (diameter 4–20 μm, sphericity 0.1–1.0, stained above a grayscale threshold 60 units below peak gray value) were analyzed using a DMRB microscope, an Olympus DP73 camera and cellSens Dimension software (version 1.8.1, Olympus Corporation). Images were collected from consecutive sections (each 120 μm apart) using a ×5 objective lens.

Cortical regions of interest were the dorsal ACC (24b), ventral ACC (24a), and prelimbic cortex (PL). The secondary somatosensory cortex (S2) was added as a control region as it has limited interconnectivity with ACC (Vogt and Miller 1983). Subcortical areas were the anterodorsal (AD), anteromedial (AM), and anteroventral (AV) thalamic nuclei, along with nucleus reuniens/rhomboid nucleus (Re/RH) and the rostral thalamic reticular nucleus at the level of the ATN. (Re/RH nuclei cannot be safely distinguished in tissue not counterstained.) The reticular nucleus was subdivided into its ventral and dorsal segments, reflecting how the ventral segment is preferentially interconnected with the ACC, whereas the dorsal segment is preferentially interconnected with retrosplenial cortex (Lozsádi 1994).

For each hemisphere in each case, 8 images were generated for the ACC and 4 for both PL cortex and the ATN. Three further images were generated for secondary somatosensory cortex as well as for the rostral reticular nucleus and Re/RH nuclei. For each case, a mean Fos count was generated for each region of interest.

## Data Analysis

### Behavior

For the first behavioral task, an initial analysis of variance (ANOVA) tested for any effects of rewarded dimension (whether rats required to attend to odor or digging media to solve the first discriminations differed), with stage (8 levels) as a within-subjects factor, and first dimension (2 levels) and group (2 levels) as between-subjects factors. Provided no main effect of rewarded dimension and no interactions between this factor and group were found, data were pooled across dimensions for all subsequent analyses.

A 2-way ANOVA examined mean trials to criterion for all 8 stages of the first attentional set-shifting task, with stage (8 levels) as a within-subjects factor and group (2 levels) as a between-subjects factor. Following significant interactions, simple main effects analysis based on the pooled-error term was used to explore group differences as well as differences between ID4 and ED to assess shift effectiveness (Howell 2010). The shift-cost was calculated as the difference between the mean trials to criterion for ID1–4 and for acquiring the ED (Chase et al. 2012). One-sample *t*-tests (2 tailed) assessed whether the shift-cost was higher or lower than 0. Errors to criterion were also recorded, giving the same pattern of results across experiments.

For the spatial ED task, an initial ANOVA checked whether the spatial dimension (the left or right chamber in which rats were rewarded) had any effects on performance. This included stage (4 levels) as a within-subjects factor, and first chamber (2 levels) and group (2 levels) as between-subjects factors. Null results allowed the data to be pooled across dimensions for all subsequent analyses. Next, a 2-way ANOVA with stage (4) as a within-subjects factor and group (2 levels) as a between-subjects factor examined group differences in mean trials to criterion. Significant interactions were investigated using simple main effects based on the pooled-error term. Shift-cost was the difference in the trials to criterion for ID and ED_spatial_. One-sample *t*-tests (2 tailed) assessed whether the shift-cost was higher or lower than 0.

All analyses used JASP computer software (version 0.11.1). Data were initially checked for normality using the Shapiro–Wilk test. Mauchly’s test for sphericity was considered and, where violated, Greenhouse–Geisser corrections were applied to the degrees of freedom. The alpha level was set at *P* < 0.05 throughout. To give an estimate of effect size, partial eta squared is reported for all significant main effects and interactions.

### Fos-Positive Cell Counts

A 2-way ANOVA examined mean Fos-positive cell counts in the cortical regions of interest, with region (3 levels, 24b, 24a, PL, S2) as a within-subjects factor and group (2 levels) as a between-subjects factor (Experiment 1 only). Similarly, a 2-way ANOVA examined counts in the ATN and Re/RH nuclei with region (4 levels, AD, AM, AV, Re/RH) as a within-subjects factor and group (2 levels) as a between-subjects factor. The cell counts in the rostral thalamic reticular nucleus (dorsal and ventral) were analyzed separately. A 2-way ANOVA assessed for group differences by region (dorsal vs. ventral reticular) as a within-subject factor as well as between-subjects factor of group. Where interactions were found between region and group, simple effects based on the pooled-error term explored group differences.

## Results

### Experiment 1: ACC ID and ED Set-Shifting

#### Histology

**Figure 1:**
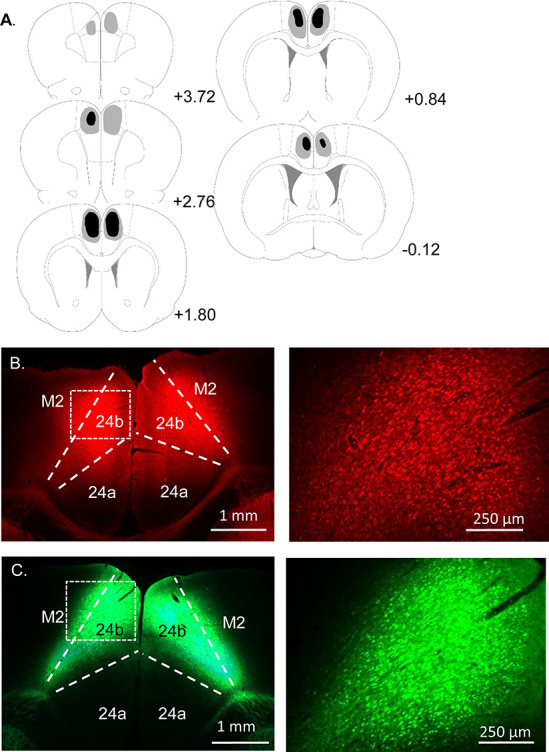
Experiment 1: Summary of virus expression in the inhibitory DREADD (hM4Di) and control groups. (*A*) Diagrammatic coronal reconstructions showing the individual cases with the largest (gray) and smallest (black) expression of mCherry in the inhibitory DREADD group. Numbers refer to the distance (mm) from bregma (adapted from Paxinos and Watson 2005). (*B*) hM4Di mCherry expression in dorsal ACC (24b). (*C*) GFP expression in the control group. All animals in both groups displayed robust virus expression centred in area 24b and its efferent targets. Other abbreviations: 24a, ventral ACC; M2, secondary motor cortex.

Two animals, one from each group, were excluded from the analysis due to a lack of expression of the virus in the ACC. In the remaining animals, analysis confirmed that both viruses were concentrated in the dorsal aspect of the ACC, area 24b, with some spread into ventral ACC, area 24a (Vogt and Peters 1981) (Fig. 1). Only limited virus reached the edges of the dorsal PL or rostral retrosplenial cortices.

#### Behavior

The initial stimulus contingency (i.e., odor or media rewarded) did not affect performance or interact with group (all *F*s <1), so consequently the data were pooled across both dimensions in all analyses.

**Figure 2:**
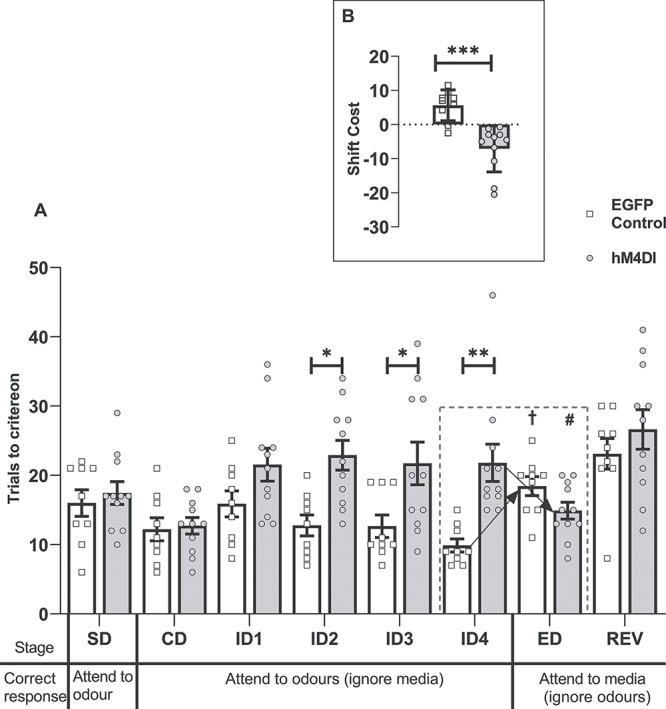
Experiment 1: Activation of inhibitory DREADDs in the ACC (hM4Di) impairs ID set-formation but facilitates ED set-shifting. (*A*) Inhibitory DREADDs in the ACC (hM4Di) impaired ID set-formation but facilitated the ED-shift. The hM4Di group required more trials to solve several individual ID stages (than the control group, ^*^P < 0.05, ^*^^*^P < 0.01). While the control group required more trials to solve the ED-shift than ID4 (†*P* < 0.001), the hM4Di group took fewer (than ID4, #*P* < 0.05; group interaction, *P* < 0.001). (*B*) ACC (hM4Di) animals shifted in fewer trials than controls (^*^^*^^*^P < 0.001, difference between mean trials to criterion for ID1–4 and ED). While controls had a shift-cost (higher than zero, *P* < 0.01), the hM4Di group had a shift-benefit (*P* < 0.01). Abbreviations: CD, compound discrimination; REV, reversal; SD, simple discrimination. Note, all discriminations were counterbalanced.

As is clear from Figure 2*A*, the hM4Di group did not form an attentional-set, requiring more trials to complete several of the ID stages yet outperforming controls at the ED-shift stage. ANOVA revealed a group by 8-stage interaction (*F*_7,126_ = 3.72, *P* = 0.001, η_p_^2^ = 0.171) as well as a main effect of group (*F*_1,18_ = 13.11, *P* = 0.002, η_p_^2^ = 0.422). Simple main effects confirmed that the hM4Di group required more trials to solve individual ID stages (ID2 [*F*_1,18_ = 6.78, *P* = 0.018], ID3 [*F*_1,18_ = 5.42, *P* = 0.032], and ID4 [*F*_1,18_ = 9.40, *P* = 0.007]) relative to controls, with overall performance across the 4 ID shifts differing by group (*F*_1,18_ = 20.03, *P* = 0.00003, η_p_^2^ = 0.527). While the controls, as expected, required more trials to solve the ED-shift than ID4 (*F*_1,18_ = 14.58, *P* = 0.001), the hM4Di group required fewer trials than for ID4 (*F*_(1,18)_ = 11.62, *P* = 0.003; interaction between stage and group *F*_1,18_ = 26.21, *P* < 0.001, η_p_^2^ = 0.593).

Set-shifting (ID to ED) was also examined by considering the shift-cost, based on the difference in mean trials to criterion across ID1–4 and trials for the ED (Chase et al. 2012; Wright et al. 2015). This analysis revealed a qualitative difference between the 2 groups (*F*_1,18_ = 23.21, *P* < 0.001, η_p_^2^ = 0.563, Fig. 2*B*). While the shift-cost of controls was higher than zero (1-sample *t*_8_ = 3.75, *P* = 0.006), the hM4Di group displayed a shift-benefit, that is, lower than zero (*t*_10_ = −3.47, *P* = 0.006). Finally, there was no evidence of a group difference on the REV condition (*F* < 1), nor was there a difference in the mean times taken to complete a trial across the task (*F* < 1).

##### Second extradimensional-shift (spatial)

**Figure 3:**
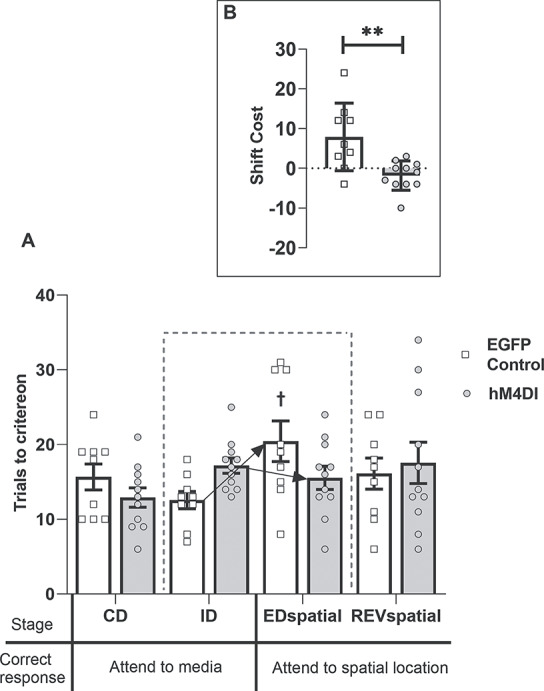
Experiment 1: Activation of inhibitory DREADDs in the ACC (hM4Di) facilitates spatial ED set-shifting. (*A*) The control group displayed a shift-cost on an ED_Spatial_, taking more trials to solve this stage than the ID stage (†*P* < 0.05). There was no difference in the number of trials taken to complete these 2 stages in the hM4Di (hM4Di) group. (*B*) The hM4Di group shifted in fewer trials than controls (^*^^*^P < 0.01). Abbreviations: CD, compound discrimination; REVspatial, spatial reversal. Note, all discriminations were counterbalanced.

Rats received a further behavioral test (Wright et al. 2015) to test the generality of the set-shifting facilitation. Now, for the first time, spatial position determined reward (Table 2). There were no group differences on the initial 2 discriminations (CD, ID), but the hM4Di group were again relatively facilitated on the subsequent dimensional switch (ED_spatial_; Fig. 3*A*). This facilitation is reflected in the interaction from ID to ED_spatial_ (*F*_1,18_ = 11.18, *P* = 0.004, η_p_^2^ = 0.383). Simple main effects analysis of this interaction confirmed that the control animals required more trials to complete the ED_spatial_ stage relative to the preceding ID stage (*F*_1,18_ = 13.94, *P* = 0.002), whereas there was no difference in the hM4Di group (*F* < 1). There was a group difference on this shift-cost measure (*F*_1,18_ = 11.78, *P* = 0.003, η_p_^2^ = 0.396). The shift-cost in controls was above zero (*t*_8_ = 2.79, *P* = 0.024; Fig. 3*B*), the hM4Di group displayed neither a cost nor benefit (*t*_10_ = −1.64, *P* = 0.13). There was no difference in mean time taken per trial between the groups (*F* < 1).

##### Fos-positive cell counts

**Figure 4:**
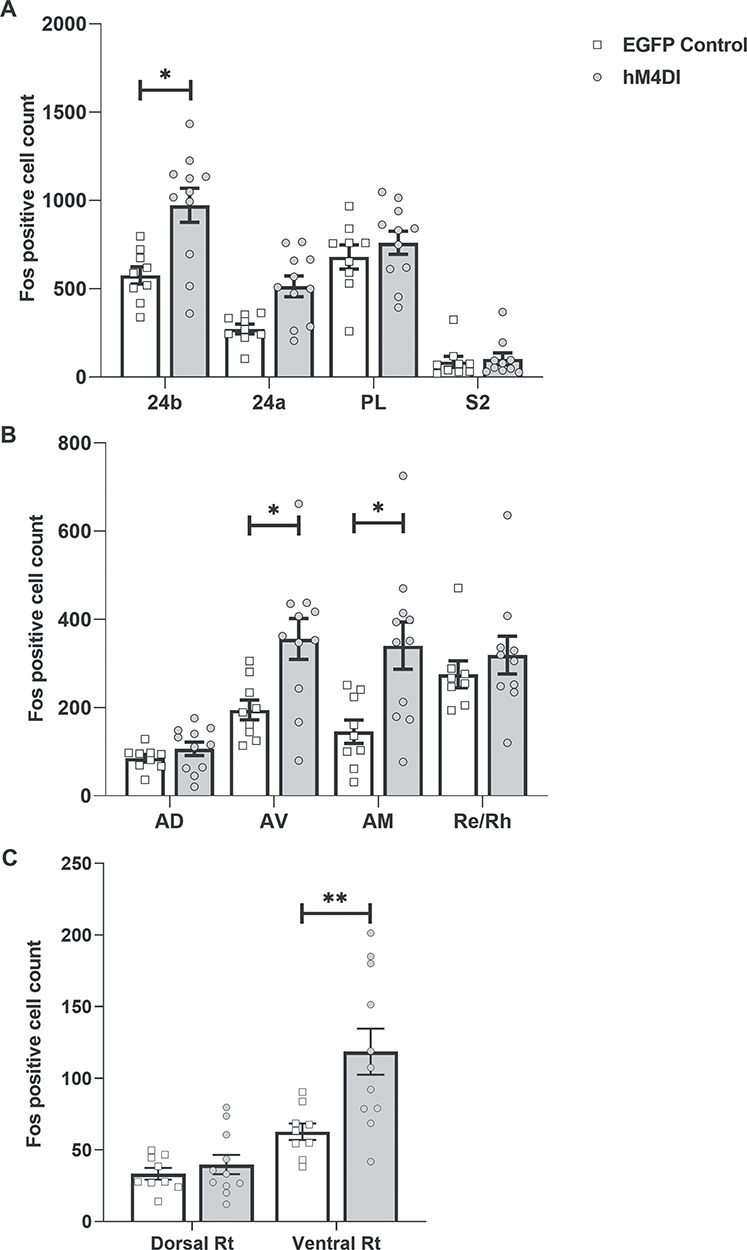
Experiment 1: Mean Fos-positive cell counts in regions of interest. (*A*) Inhibitory DREADDs in the ACC (hM4Di) resulted in higher Fos counts in area 24b, the center of the injection sites. (*B*) The hM4Di group showed an elevation in those anterior thalamic sites receiving dense inputs from the ACC. (*C*) A relative increase in Fos counts was present in the ventral, but not dorsal, thalamic reticular nucleus, corresponding to the differential anterior cingulate inputs to this region. Abbreviations: 24a, ventral anterior cingulate cortex; 24b, dorsal anterior cingulate cortex; PL, prelimbic cortex; S2, secondary somatosensory cortex; AD, anterodorsal thalamic nucleus; AV, anteroventral thalamic nucleus; AM, anteromedial thalamic nucleus; Re/Rh reuniens and rhomboid nuclei; Rt, thalamic reticular nucleus. ^*^P < 0.05, ^*^^*^P < 0.01.

In the inhibitory DREADD group, higher Fos-positive cell counts were observed in cortical area 24b (*F*_1,18_ = 7.56, *P* = 0.013), but not area 24a (*F*_1,18_ = 2.82, *P* = 0.11), PL cortex (*F* < 1), or secondary somatosensory cortex (group by region interaction [*F*_(3,54)_ = 9.63, *P* < 0.001, ή ^2^ = 0.349]) (Fig. 4*A*). Within subcortical sites (Fig. 4*B*), higher Fos-positive cell counts were again found in the inhibitory DREADD group in both the AV (*F*_1,18_ = 6.71, *P* = 0.018) and AM thalamic nuclei (*F*_1,18_ = 4.57, *P* = 0.046), but not the AD thalamic nuclei or Re/RH nuclei (*F* < 1), (group by region interaction [*F*_3,51_ = 5.03, *P* < 0.01, ή^2^ = 0.228]). For the reticular thalamic nucleus (Fig. 4*C*), increased Fos counts were found in the ventral (*F*_1,18_ = 8.88, *P* = 0.008), but not the dorsal (*F* < 1)(group by region interaction [*F*_2,36_ = 19.1, *P* < 0.001, ή^2^ = 0.52)] part of this nucleus. The Supplementary Data provide images of Fos-positive cells in various regions of interest.

### Experiment 2: ACC Efferents to the ATN and ID and ED Set-Shifting

#### Histology

**Figure 5:**
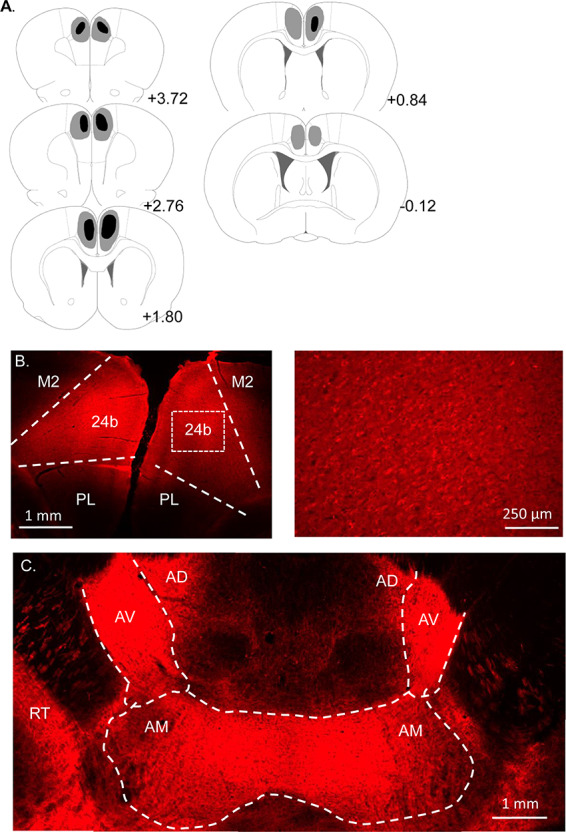
Experiment 2: Summary of virus expression in the inhibitory DREADD (hM4Di) group. (*A*) Diagrammatic coronal reconstructions showing individual cases with largest (gray) and smallest (black) expression of mCherry in the inhibitory DREADD group. Numbers refer to the distance (mm) from bregma (adapted from Paxinos and Watson 2005). (*B*) hM4Di mCherry expression in dorsal ACC (24b). (*C*) hM4Di mCherry expression in the AM and AV thalamic nuclei. All animals in both groups had robust virus expression in area 24b and its efferent targets. Note: GFP expression in the control group was comparable to the inhibitory DREADD group in Experiment 1 (Fig. 2). Other abbreviations: M2, secondary motor cortex; PrL, prelimbic cortex; Rt, reticular thalamic nucleus.

In both groups, all animals displayed robust virus expression in dorsal ACC, area 24b, with some spread into ventral ACC, area 24a (Fig. 5*A*,*C*).

There was only limited spread to the edges of the dorsal PL or rostral retrosplenial cortices. Both viruses showed extensive anterograde transport to sites that receive direct inputs from the ACC. Among these sites there was dense label in the AM thalamic nucleus and subregions of the AV thalamic nucleus (Fig. 5*D*).

**Figure 6:**
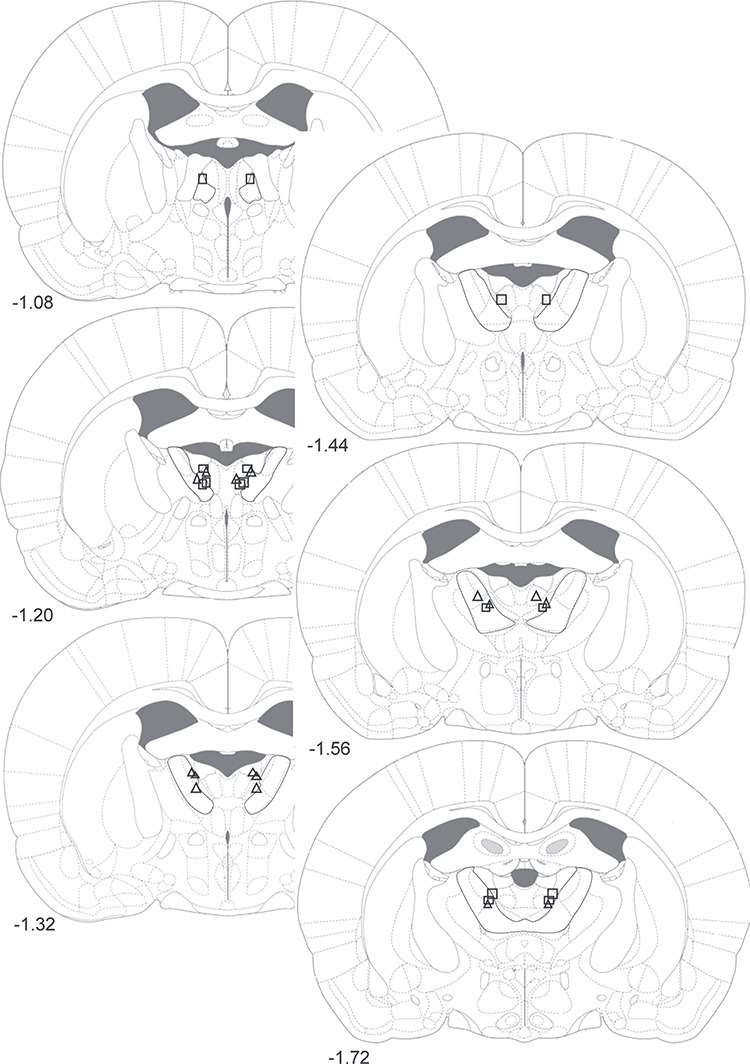
Experiment 2: Summary of thalamic cannulae placements in the inhibitory DREADD group and control groups. Diagrammatic reconstructions showing the locations of tips of cannulae aimed at the ATN (demarcated with black lines). Triangles represent cases from the inhibitory DREADD group and rectangles represent cases from the control group. Numbers refer to the distance (mm) from bregma (adapted from Paxinos and Watson 2005).

Two animals, one from each group, had cannula tips located outside of the target region of the ATN and were excluded from analysis. Figure 6 illustrates the location of the cannulae tips in the remaining animals, identified by histological analysis. The injectors had a 2-mm projection, resulting in infusion locations approximately 2 mm ventral to the tips of the cannulae.

#### Behavior

The initial stimulus contingency (odor or media rewarded) did not affect overall performance or interact with group (max *F*_(1,12)_ = 1.36, *P* = 0.27), so the data were pooled across the 2 dimensions in the analysis.

**Figure 7:**
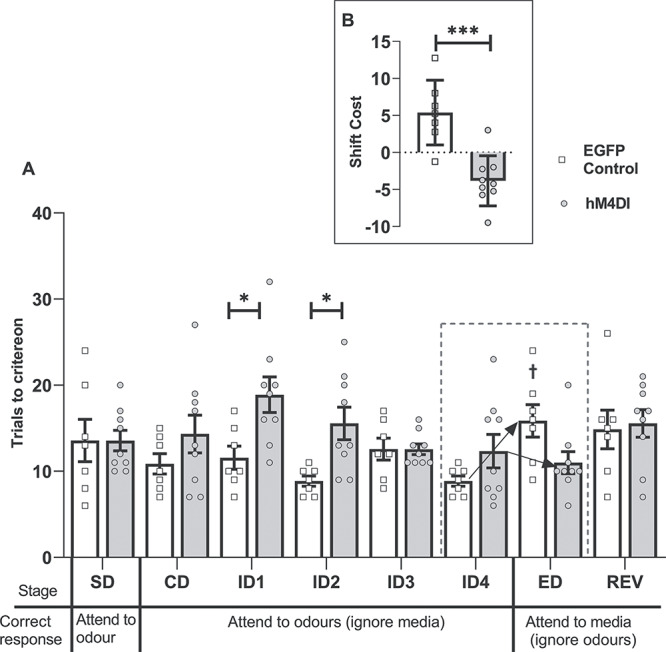
Experiment 2: DREADD-mediated disruption of ACC efferents to the ATN (hM4Di) impairs ID set-formation and facilitates ED set-shifting. (*A*) The hM4Di animals were impaired at some ID discriminations (^*^P < 0.05) but facilitated the ED-shift. While controls required more trials to solve ED than ID4 (†*P* < 0.01), the hM4Di group did not (group interaction, *P* < 0.01). (*B*) hM4Di animals shifted faster than controls (^*^^*^^*^P < 0.001, difference between mean trials to criterion for ID1–4 and ED). While controls had a shift-cost (higher than zero, *P* < 0.05), the hM4Di group had a shift-benefit (*P* < 0.01). Abbreviations: CD, compound discrimination; REV, reversal; SD, simple discrimination.

Inspection of Figure 7*A* reveals that the 2 groups differed on several stages of the task. ANOVA yielded a significant main effect of group (*F*_1,14_ = 5.39, *P* = 0.036, η_p_^2^ = 0.278) as well as an interaction between group and the 8 discrimination stages (*F*_7,98_ = 2.93, *P* = 0.008, η_p_^2^ = 0.173, Fig. 7*A*). Simple main effects analyses showed that activation of DREADDs disrupted learning of ID1 and ID2 (min *F*_1,14_ = 6.87, *P* = 0.02), but there were no differences between the groups on either ID3 or ID4 (max *F*_1,14_ = 1.85, *P* = 0.19) indicating that the hM4Di group was initially slower to solve ID-shifts, but this did not fully preclude attentional-set formation. However, further analysis showed average performance across the 4 ID shifts differed by group (*F*_1,14_ = 14.09, *P* = 0.002, η_p_^2^ = 0.502).

Nevertheless, ED learning was facilitated in the hM4Di group (group interaction ID4, ED, *F*_1,14_ = 9.72, *P* = 0.008, η_p_^2^ = 0.41). As expected, controls took more trials to solve the ED (*F*_1,14_ = 12.19, *P* = 0.004) than the preceding ID4, whereas simple main effects analysis showed no difference in the number of trials taken to complete these task stages in hM4Di group (*F* < 1).

Additional comparisons based on mean trials to criterion for ID1–4 and ED (shift-cost) confirmed these group differences (*F*_1,14_ = 22.77, *P* < 0.001, η_p_^2^ = 0.619) (Fig. 7*B*). As expected, the shift-cost in controls was above zero (*t*_6_ = 3.26, *P* = 0.017). In contrast, the hM4Di group showed a shift-benefit, that is, less than zero (*t*_8_ = −3.40, *P* = 0.009). Finally, performance on the REV task did not differ (*F* < 1) between groups (Fig. 7*A*).

##### Second extradimensional-shift (spatial)

**Figure 8:**
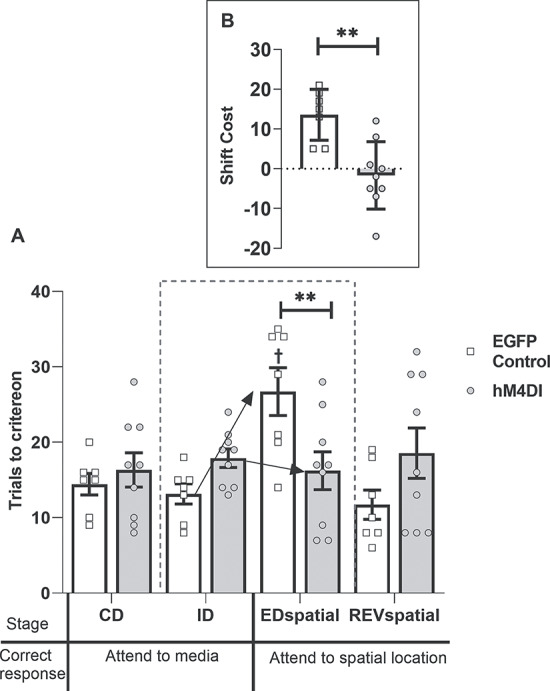
Experiment 2: DREADD-mediated disruption of ACC efferents to the ATN (hM4Di group) facilitates spatial ED set-shifting. (*A*) The control group displayed a shift-cost on the ED_spatial_, taking more trials to solve this discrimination than the ID stage (†*P* < 0.001). In the hM4Di group, there was no difference in the number of trials taken to complete these 2 stages. (*B*) The hM4Di group shifted faster than controls (^*^^*^P < 0.01). Abbreviations: CD, compound discrimination; REVspatial, spatial reversal. Note, all discriminations were counterbalanced. Note, all discriminations were counterbalanced.

As in Experiment 1, rats received a second behavioral test including a discrimination where spatial position, for the first time, determined reward (Table 2). There were no group differences on the initial 2 discriminations (CD, ID), but the hM4Di group were again relatively facilitated on the subsequent dimensional switch (ED_spatial_, Fig. 8*A*). This facilitation is reflected in the interaction from ID to ED_spatial_ (*F*_1,14_ = 15.58, *P* = 0.001, η_p_^2^ = 0.527). Simple main effects analysis of this interaction confirmed that the control group required more trials to complete the ED_spatial_ stage relative to the preceding ID stage (*F*_1,14_ = 21.97, *P* = 0.0003), whereas there was no difference in performance across these 2 stages in the hM4Di group (*F* < 1). There was also a simple main effect of group on the ED_spatial_ stage (*F*_1,14_ = 9.39, *P* = 0.0008).

Further analysis (Fig. 8*B*) showed a group difference on the shift-cost measure (*F*_1,14_ = 9.35, *P* = 0.009, η_p_^2^ = 0.40). The shift-cost in controls was above zero (*t*_6_ = 5.61, *P* = 0.001), whereas the hM4Di group displayed neither a cost nor benefit (*t* < 1). There was no difference in mean time taken per trial between the groups (*F* < 1).

## Discussion

The present study began by demonstrating that the ACC is required for attentional-set formation in rats. The inhibitory ACC DREADD group failed to show accelerated learning over successive ID-shifts and was impaired relative to control animals on several of these discriminations. However, when first required to solve a discrimination involving the previously irrelevant stimulus dimension (ED), the same animals showed a significant shift-benefit, requiring fewer trials to solve the ED relative to the proceeding ID stages. This profile, impaired attentional-set formation but facilitated switching, reproduces precisely the pattern of performance on the same task by rats with lesions in the ATN (Wright et al. 2015). Experiment 2 then showed how interactions between the ACC and ATN are required for these processes, as chemogenetic disruption of ACC efferents to the ATN attenuated early attentional-set formation but again facilitated ED shifts.

### Methodological Considerations

A primary consideration relates to whether the administration of clozapine in the EGFP control animals had off-target or nonspecific effects that could confound interpretation of the behavioral results (Roth 2016; Smith et al. 2016). This issue is particularly germane to Experiment 1 that involved systemic injections of the ligand. Although the possibility of off-target effects of clozapine cannot entirely be discounted, there is little evidence to suggest that such effects influenced the key behavioral findings. For example, comparing the performance of the EGFP (control) groups across Experiments 1 and 2, there was no difference in the magnitude of the key shift-cost measure, even though clozapine was only administered systemically in Experiment 1. Furthermore, the profile of task performance of the EGFP groups (with clozapine) in the current experiments very closely match those of controls groups (without clozapine) previously reported both by ourselves and by other groups (Chase et al. 2012; Lindgren et al. 2013; Wright et al. 2015; Powell et al. 2017; Tait et al. 2018). The unique learning profile of the experimental groups (impaired ID but facilitated ED shift performance) is similarly difficult to reconcile with an account framed in terms of off-target effects of the ligand.

In addition to the behavioral effects of DREADD activation, we also assessed levels of Fos protein within the dorsal ACC and its key efferent targets (Fig. 4). Two informative patterns of results emerged. The first was that changes in Fos-positive cell counts were concentrated in 24b and in specific targets of the ACC (Lozsádi 1994; Shibata and Naito 2005), namely the AM and AV thalamic nuclei, as well as the ventral part of the thalamic reticular nucleus (Fig. 4). Meanwhile, the PL and somatosensory cortices, alongside thalamic sites that receive much lighter ACC inputs (the AD thalamic nucleus, the dorsal part of the thalamic reticular nucleus, Re/RH nuclei) (Lozsádi 1994; Vertes 2002; Shibata and Naito 2005), did not show significant Fos changes. These results highlight the likely selectivity of DREADD activation in ACC and its efferents, as well as the particular significance of those thalamic nuclei targeted in Experiment 2.

The second pattern was less expected: clozapine increased, rather than decreased, Fos levels in these same sites (Fig. 4 and Supplementary Fig. S1). While studies assessing the effects of inhibitory DREADD activation on network activity have generally reported decreased c-*fos* expression (Koike et al. 2016; Choi and McNally 2017), increased activity has occasionally been seen (López et al. 2016). Two related factors might explain the present pattern. The first concerns the dense reciprocal connections between ACC and the ATN. The second is that anterior cingulate excitatory neurons preferentially innervate the more ventral rostral thalamic reticular nucleus, which sends inhibitory projections to the ATN as well as receiving excitatory inputs from the same nuclei (Lozsádi 1994; Gonzalo-Ruiz and Lieberman 1995; Zikopoulos and Barbas 2006). At this stage we can only speculate that the disruption of reticular gating may have disinhibited anterior thalamic–cortical circuitry. Following on from this, it is arguably better to regard the inhibitory DREADD action as disrupting rather than “silencing,” which may help to explain the clear parallels between the ACC DREADD group and the effects of lesions within the ATN (Wright et al. 2015).

### ACC, the ATN, and Attentional-set formation

While it has previously been reported that lesions in the orbitofrontal cortex and dorsomedial striatum impair attentional-set formation (Chase et al. 2012; Lindgren et al. 2013), the deficit in attentional-set formation combined with the facilitation of ED shifts seen here after inhibition of the ACC (Experiment 1) and its efferents to the ATN (Experiment 2) is highly unusual. This profile of impaired ID but facilitated ED shifting cannot be explained by a general deficit in discrimination learning or because the inhibitory DREADD groups attended to both perceptual dimensions equally, as otherwise the ED shift should have been acquired at the same rate as the ID shifts. Rather, this learning profile is consistent with an initial bias to process poor predictors of reward. Such a learning bias would retard ID shifts when animals are required to preferentially attend to one stimulus dimension that is consistently predictive of reward (e.g., odor) while ignoring other irrelevant stimulus dimensions (e.g., media). Attending to the irrelevant stimulus dimensions would not only disrupt the formation of an attention set but also facilitate acquisition of the ED when that previously irrelevant dimension becomes relevant to solving the discrimination. Over the preceding series of ID shifts, normal animals learn to ignore the partially reinforced dimension and instead attend to the dimension that is the most reliable predictor of reinforcement (Mackintosh 1975); a process that drives ID performance at the expense of ED shifts. In contrast, disrupting ACC activity (Experiment 1) or the flow of information from the ACC to the ATN (Experiment 2) produced the opposite profile of performance. The implication is that activation of DREADDS within the ACC or ACC terminals in the ATN disrupted the normal process whereby attention to a stimulus increases if it is the best predictor of reinforcement and, as a corollary, decreases to poor predictors of reinforcement.

This interpretation, an abnormal attentional bias toward poor predictors of reward, was strongly supported by the facilitation of learning seen in the DREADD groups at the ED stage when the hitherto irrelevant dimension now became critical to solving the discrimination. Moreover, the experiments also highlight the generality and robustness of these attentional effects as set-shifting was also assessed for another stimulus class, spatial position. As expected, controls animals took longer to solve a discrimination involving the hitherto irrelevant spatial position of the reward, whereas the experimental groups did not show a shift-cost. Although this effect is more nuanced than that seen on the original ED shift, as the experimental animals did not show a shift-benefit (i.e., fewer trials to criterion relative to their performance on the preceding ID stage), the group difference on the shift-cost measure again indicates facilitated shifting relative to the control animals.

Meanwhile, the selectivity of the current DREADDs effects was underlined by repeated evidence of intact REV learning (Experiments 1 and 2), where reward contingencies switch but remain within the same stimulus pair, so modality stays neutral. REV deficits are, however, seen after orbitofrontal cortex lesions (Dias et al. 1996a, 1996b; Chase et al. 2012) (but see Murray and Rudebeck 2018). These dissociable profiles highlight how “cognitive flexibility” reflects multiple processes (Kehagia et al. 2010).

Experiment 2 targeted ACC projections to the ATN. The more selective nature of this manipulation helps to explain the less disruptive effects on ID1–4 than those seen in Experiment 1, in which multiple anterior cingulate efferents were compromised. While disrupting information flow from the ACC to the ATN initially appeared to attenuate attentional-set formation, by the final 2 ID shifts there were no differences in performance between the 2 groups. However, overall performance across the 4 ID stages was impaired relative to the control groups. One potential implication is that this more selective manipulation retarded rather than precluded attentional-set formation. Nevertheless, despite the apparent greater selectivity of this effect in Experiment 2, ED set-shifting was still facilitated, as a significant “shift-benefit” was again observed. The implication is that the prior attenuation in attentional-set formation was sufficient to produce enhanced ED shift performance. These results advance evidence that the ATN have an important role in set-formation (Wright et al. 2015), evidence that includes neuropsychological findings (de Bourbon-Teles et al. 2014). This attention role is, however, specific as ATN lesions spare vigilance tasks (Chudasama et al. 2001), despite such tasks being disrupted by ACC lesions (Muir et al. 1996). In turn, these ACC lesion attentional effects are qualitatively different from those associated with the PL and infralimbic cortices (Muir et al. 1996; Koike et al. 2016; Fisher et al. 2019).

The demonstration here that the ACC and its efferents to the ATN mediate attentional-set formation accords with evidence demonstrating a role for the ACC in incorporating reward history with current contingencies to determine action selection (Behrens et al. 2007; Rushworth et al. 2007; Shenhav et al. 2016). For example, lesions in the primate ACC impair the ability to use the past history of actions and their outcome to select appropriate behavioral responses, whereas neurons in the primate ACC respond during the generation of exploratory actions and the monitoring of outcomes of these actions (Hayden and Platt 2010). If one function of the ACC is to mediate the relationship between recent actions–outcomes and current behavioral choices (Rushworth et al. 2004, 2007; Quilodran et al. 2008), ACC disruption would be predicted to impair animals’ ability to establish those stimulus-dimensions consistently associated with reward, and with no-reward, respectively. This description matches the pattern of behavior seen in the current experiments. A failure to acquire an attentional-set would be expected to abolish the shift-cost normally seen at the ED stage (Durlach and Mackintosh 1986). Inhibition of the ACC and its efferents to the ATN did not just abolish the shift-cost associated with the ED, it facilitated switching. The implication, therefore, is that the rodent ACC in concert with the ATN normally mediates the relationship between previous actions that are reliably associated with outcomes and current behavioral choices.

## Conclusion

This facilitation of set-shifting after ACC disruption is the converse of that seen after medial prefrontal cortex lesions. While lesions of medial frontal areas in monkeys and rats spare set-formation, ED switching is protracted, requiring more trials than controls (Dias et al. 1996a; Dias et al. 1996b; Birrell and Brown 2000). Likewise, patients with frontal lobe damage have difficulty in shifting to a previously irrelevant dimension (Owen et al. 1991). The resulting double dissociations (ACC vs. medial prefrontal) on set-formation and set-switching can best be explained by 2 competing processes (Mackintosh 1975; Pearce and Hall 1980; Pearce and Mackintosh 2010), each with distinct neural underpinnings. While ACC activity would normally promote adherence to a previously successful stimulus class (lost after ACC inhibition), medial prefrontal cortex activity promotes attentional switching (Sharpe and Killcross 2014).

These processes match those separately predicted by learning theorists (Mackintosh 1975; Pearce and Hall 1980; Pearce and Mackintosh 2010). Together, they determine how past learning guides present choice behavior (Pearce and Mackintosh 2010). It is presumed that the normal activity of the rat ACC, in cooperation with the ATN, is to focus learning and attention on successful reward outcomes while updating internal models of the environment, for example, ID set-formation. Disruption of this function leads to an initial excessive attention to irrelevant cues that, paradoxically, can facilitate learning when contingencies change. The interplay between these “switch” and “stay” processes closely relates to cognitive inflexibility, a prominent feature of multiple psychiatric conditions. The specific difficulty of selecting appropriate stimuli to guide choice behavior has obvious similarities with the symptoms of psychiatric conditions, including depression, schizophrenia, and autism spectrum disorders (Gold et al. 1997; Merriam et al. 1999; Tsuchiya et al. 2005; Meiran et al. 2011), all conditions that display ACC dysfunction (Yücel et al. 2003). One such example concerns how attention to irrelevant cues is related to positive symptoms in schizophrenia (Morris et al. 2013).

Finally, these findings have implications for our understanding of the contribution of thalamocortical and corticothalamic circuits to cognition. Recent work has consistently shown how accounts of thalamic function in terms of a relay station to cortex are no longer tenable (Mitchell et al. 2014; Crandall et al. 2015; Bolkan et al. 2017; Guo et al. 2017; Alcaraz et al. 2018; Wolff and Vann 2019). The current findings provide causal evidence for the involvement of ACC–ATN pathways in driving attention to task-relevant information, consistent with an integrative role of these circuits in cognition. It remains to be established how information flow from the ATN to the ACC contributes to these effects. A related consideration is whether distinct ATN are differentially involved in these attentional processes.

## Supplementary Material

Supplementary_Figure_1_bhaa353

ACC_ATN_Supplementary_Materials_bhaa353

## Notes

We thank E. Amin for her technical support. *Conflict of Interest*: None declared.
