# Supplementary figures and images for: Chemogenetics Reveal an Anterior Cingulate–Thalamic Pathway for Attending to Task-Relevant Information
Source: Cereb Cortex. 2020 Nov 30;31(4):2169–86. doi: 10.1093/cercor/bhaa353 (PMC7945017; doi:10.1093/cercor/bhaa353)

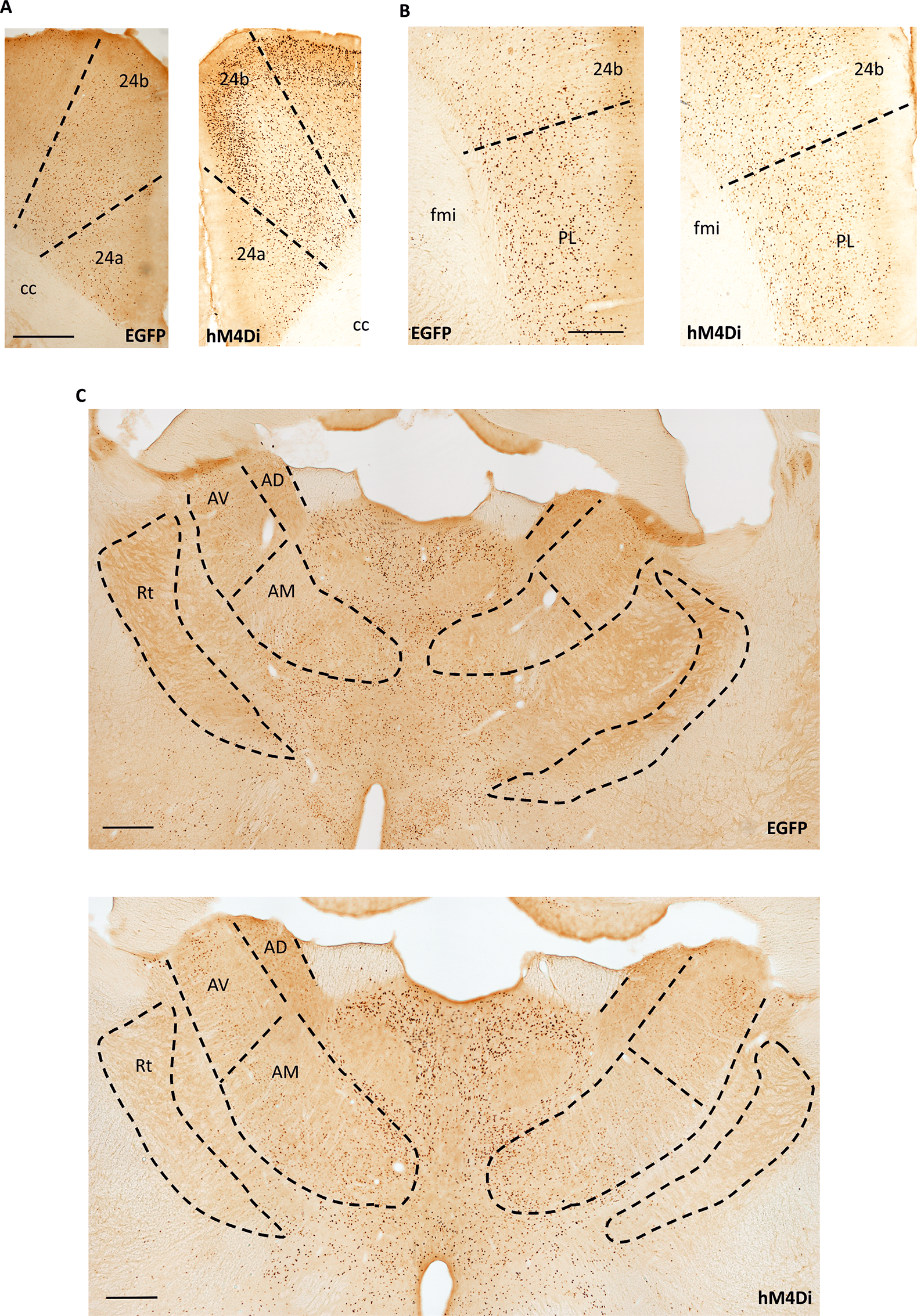

Supplement: Supplementary_Figure_1_bhaa353 [file supplementary_figure_1_bhaa353.png]
