# Supplementary material for: Chemogenetics Reveal an Anterior Cingulate–Thalamic Pathway for Attending to Task-Relevant Information
Source: Cereb Cortex. 2020 Nov 30;31(4):2169–86. doi: 10.1093/cercor/bhaa353 (PMC7945017; doi:10.1093/cercor/bhaa353)
Supplement: ACC_ATN_Supplementary_Materials_bhaa353 [file acc_atn_supplementary_materials_bhaa353.docx]

**Supplementary Data**

**Contents**

Supplementary Figure Caption……………………………………………………………………………….1

Supplementary Figures…………………………………………………………………………………………2/3

**Supplementary Figure 1 - Fos-positive cell counts following activation of inhibitory DREADDs in areas 24b (Experiment 1).**

Photomicrographs of Fos-positive nuclei in the control (EGFP) and DREADD (hM4Di) groups in area 24b (A), prelimbic cortex (B) and rostral thalamus (C). Abbreviations: 24b, dorsal anterior cingulate cortex; AD, anterodorsal thalamic nucleus; AV, anteroventral thalamic nucleus; AM, anteromedial thalamic nucleus; cc, corpus callosum; fmi, forceps minor of the corpus callosum; PL, prelimbic cortex; Rt, thalamic reticular nucleus. Scale bars represent 500 µm.
